# Temporalis Muscle Changes Following Botulinum Toxin A Injections in Masseter Hypertrophy Patients: A Randomized Triple-Blinded Trial

**DOI:** 10.1007/s00266-024-04064-4

**Published:** 2024-05-13

**Authors:** Bryanne B. de Souza Nobre, Luciana de Oliveira Resende Machado, Rodrigo Lorenzi Poluha, Mariana Barbosa Câmara-Souza, Ana Claudia Carbone, Andre Mariz de Almeida, Anastasios Grigoriadis, Abhishek Kumar, Giancarlo De la Torre Canales

**Affiliations:** 1 Department of Dentistry, Ingá University Center, Uningá, Paraná, Brazil; 2 Department of Dentistry, State University of Maringa, Paraná, Brazil; 3 Private Practice, São Paulo, Brazil; 4 Egas Moniz Center for Interdisciplinary Research (CiiEM), Egas Moniz School of Health and Science, Caparica, Almada, Portugal; 5 Division of Oral Rehabilitation, Department of Dental Medicine, Karolinska Institutet, Alfred Nobels Allé 8, Huddinge, Stockholm, Sweden; 6 Academic Center for Geriatric Dentistry, Stockholm, Sweden

**Keywords:** Botulinum toxin type A, Masseter hypertrophy, Temporalis muscle

## Abstract

**Background:**

This study aimed to elucidate the effects of botulinum toxin A (BoNT-A) treatment for patients diagnosed with masseter hypertrophy on the temporalis muscle, with a particular focus on assessing alterations in muscle thickness, electromyographic (EMG) activity, and the development of muscle pain.

**Methods:**

The present randomized triple-blinded clinical trial enrolled 26 female participants aged between 25 and 50 years complaining about masseter hypertrophy. Participants received 75U of BoNT-A (abobotulinumtoxinA) in both masseter muscles and after three months were randomized to receive a second treatment session of saline solution (S-BoNT-A) or BoNT-A (M-BoNT-A). Longitudinal assessments included temporalis muscle thickness through ultrasound, EMG activity, subjective pain, and masseter prominence severity after one, three, and six months of the first injection session. Muscle thickness, EMG, and subjective pain were analysed using two-way ANOVA with repeated measures and post hoc Sidak test, and for masseter prominence severity, Friedman and Mann–Whitney tests were used.

**Results:**

Regarding inter-group comparisons, a higher muscle thickness (*p* < 0.02) and a higher EMG activity (*p* < 0.01) were found in the M-BoNT-A group at the 6-month follow-up. For subjective pain assessments, inter-group comparisons showed a higher prevalence of painful regions in M-BoNT-A group at the 6-month follow-up (*p* < 0.02). No significant differences were found in masseter prominence severity at the 6 months assessment between groups.

**Conclusion:**

BoNT-A treatment for masseter hypertrophy lead to structural and functional changes in the temporalis muscle, presenting higher changes after multiple injections of this treatment.

**Level of Evidence I:**

This journal requires that authors assign a level of evidence to each article. For a full description of these Evidence-Based Medicine ratings, please refer to the Table of Contents or the online Instructions to Authors www.springer.com/00266.

## Introduction

Masseter hypertrophy (MH), characterized by an abnormal enlargement of the masseter muscle, significantly impacts facial aesthetics and functionality. This condition, often idiopathic in nature, can lead to facial asymmetry and psychological distress due to altered appearance [1]. Clinically, MH is associated with increased muscle stiffness and may contribute to masticatory discomfort [2]. The implications of MH extend beyond cosmetic concerns, affecting psychological well-being and overall quality of life, emphasizing its relevance in both medical and psychological domains [1].

As a minimally invasive approach for treating MH, botulinum toxin A (BoNT-A) injections have recently become more popular. The technique is preferred for its effectiveness in enhancing facial contours and diminishing muscle size. Typically, the outcomes of such treatments are observed to persist for several months, offering a balance between efficacy and minimal invasiveness [3]. Although BoNT-A is a more conservative approach compared to surgical procedures, it is not without its own risks and potential adverse effects [4]. The primary effect of BoNT-A on the masseter muscle is to diminish its size, potentially causing alterations in the function and/or size of other masticatory muscles, especially the temporalis muscle. Since the masseter and temporalis muscles work in conjunction during mandibular movements and function, changes in one can affect the other [5]. It may also generate a compensatory increase in function, increasing muscle volume and thickness, which may affect aesthetics and functionality [6, 7]. These changes should be considered as adverse effects of BoNT-A treatment for MH.

In addition, as far as we know, there is only one clinical trial assessing the changes in temporalis muscle volume in patients with MH, treated with incobotulinumtoxinA injections in masseter muscles [8]. The findings indicated a notable reduction in masseter prominence and a significant increase in temporalis volume [8]. Since muscle size and volume are dependent on muscle contraction [9], it is expected that when masseter muscles are paralysed, temporalis muscles will exhibit higher electromyographic activity. Consequently, it could be hypothesized that muscle fatigue and even muscle pain could be experienced in temporalis muscles in patients with MH treated with BoNT-A. It is important to note that, no study has assessed the hall spectrum of BoNT-A adverse effects as a treatment for MH in other masticatory muscles. Thus, it would be clinically noteworthy to assess whether the thickness, electromyographic activity, and pain in temporalis muscle increase after BoNT-A injections in masseter muscles. Additionally, because it has been reported that the effects of a single injection of BoNT-A in MH last for three to four months [8], and that muscle thickness reduces with BoNT-A doses and number of applications [10, 11], assessing the effects of single and multiple injections of this treatment is also valid.

Therefore, this study aimed to assess changes in both muscle thickness and electromyographic activity of the temporalis muscles in patients treated with single and multiple injections of BoNT-A for MH.

## Methods

This research is a secondary analysis of a study assessing BoNT-A effects on MH, which was approved by the Research Ethics Committee of Uningá University (CAAE: 63135022.3.0000.5220) and by the Brazilian Registry of Clinical Trials (RBR-7tdjcn5 - 24/01/2024). The main focus of the present study was assessing the changes of treating MH with BoNT-A on temporalis muscles. Participants, having been fully briefed on the study's aims, gave their written consent to participate in the clinical trial, conducted at the Brazilian Dental Association in Goiânia (ABO-Goiás) dental clinic from March 2, 2023, to October 17, 2023. The study was designed as a longitudinal, randomized, triple-blinded, and placebo-controlled trial, conforming to the Helsinki Declaration. The reporting of data followed the CONSORT checklist.

### Participants

The study's sample consisted of Brazilian women aged 25 to 50 years complaining about lower third facial enlargement due to MH, ranked at levels 2 to 5 on the Merz ten-point photonumeric masseter prominence scale [12]. Exclusions were made for those who previously received BoNT-A injections, facial fillers, facial surgeries, or had missing teeth, autoimmune/neuromuscular diseases, pregnancy/breastfeeding, or recent vaccinations. For sample size calculation, it was considered an average change of masseter muscle thickness of 25% (±8%), reported by a previous study [13]. Power calculation showed that with these data, 9 patients per group would demonstrate a *β* = 0.9 and *α* = 0.01. Anticipating potential dropouts due to the study’s longitudinal design, four additional participants per group were included. This resulted in a total of 26 patients, divided into two groups of 13 each. In the first group, participants received BoNT injections only once, followed by a second injection of saline (S-BoNT-A). Meanwhile, the second group (M-BoNT-A) received two BoNT injections, with the second one (BoNT) administered three months after the first.

### Study Protocol

During the study, participants underwent a series of five assessments. The first visit involved screening for eligibility and informing participants about the study's protocol and treatments. Also, participants were informed that two injection sessions would be performed, separated by a three-month interval, and that in the second injection session they will receive a second injection of BoNT-A or an injection of saline solution. In the first session, BoNT-A was injected in all participants, and in the second session after randomization, either BoNT-A or a saline solution was injected. Researchers and volunteers were not aware of the administrated treatment in the second injection session. At the second visit, baseline assessments and the first BoNT-A injection were done. Follow-up visits occurred at one month (Visit 3), three months (Visit 4) where patients were randomly assigned to receive saline solution (S-BoNT-A; *n* = 11) or another injection of BoNT-A (M-BoNT-A; *n* = 12), and six months (Visit 5) after the initial injection.

### Randomization and Blinding

Randomization was executed using an online computer program, (http://www.randomization.com/) using blocks of four patients. A technician, uninvolved in other study procedures, prepared sealed envelopes, which contained randomized treatment notes (BoNT-A or Saline solution) for each patient. The block size and randomization were unknown to the researcher injecting the substances (L.R.) the researcher examining the patients (B.B.S.N), as well as to the patients. The envelopes were opened before injections by a third researcher (A.C.C) solely in charge of syringe preparation. Then, the patient code was written on the syringes and left in the clinic before the researcher in charge of applying injections (L.R) and the patient entered. Importantly, BoNT-A and saline solution are visually indistinguishable, ensuring blinding in treatment administration.

### Interventions

BoNT-A vials were reconstituted (Dysport (abobotulinumtoxinA) 500U Ipsen, Wrexham, UK) with 2 mL of 0.9% isotonic sterile saline solution, giving a dose of 25U/0.1 mL. A total of 75U was administered in each masseter muscle into 3 injection points (25U each) bilaterally, totalling 150U for each patient. Also, a total of 0.6 mL of saline solution (0.3 mL/side) served as control. Injections were conducted in a secure area defined by a line from the tragus to the corner of the mouth, a second line 0.5 cm above the lower edge of the mandible, and within two vertical lines marking the anterior and posterior belly of the masseter muscle after a functional test (teeth clenching). Both the BoNT-A and saline solution were prepared by a trained researcher. The injections were administered by an experienced specialist in injectable facial aesthetic, not involved in any other study procedures. Injections were done using 1-ml syringes with 13 x 0 x 0.26 (26G) needles.

### Outcomes

Patients' outcomes were assessed at baseline and after 1, 3, and 6 months of the first injection session. Variables assessments were conducted at each evaluation period by a researcher (B.B.N) not involved in any other study procedures. The subjective assessments were performed using the ten-point photonumeric masseter prominence scale—Merz [12] and the Visual Analogue Scale. Objective assessments encompassed muscle thickness measured with ultrasound (US) and muscle electric activity measured with electromyography (EMG). Muscle thickness was considered as the primary outcome.

#### Ultrasound

The thickness measurement of the temporalis muscles during maximum voluntary contraction (MVC) was taken using real-time ultrasound (A6-Ultrasound Machine—transducer model L745 with a standard linear array (40 mm) *p* 11.0-5.0 MHz, SonoScape, China) by a single calibrated operator (intra-observer agreement Kappa = 0.86, for two measurements on the same cases (*n* = 3), using US imaging). The location in which muscle thickness measurements were taken was determined by palpation, after functional test (muscle clenching). Measurements were taken on the instrument’s screen (with an accuracy of 0.01 mm), corresponding to the most voluminous part of the muscle image. The average of the three measurements was considered for statistical analysis [14].

#### Electromyography

To measure the EMG signal in the temporalis muscles, a 4-channel EMG system was used (Miotool NG USB ®, Porto Alegre, RS, Brazil, frequency range: 10–700 Hz; sampling rate 3000/s; resolution: 2.44 V/bit) by a calibrated operator. Bipolar surface electrodes (Ag-AgCl discs, Covidien llc, Quebec, Canada) were placed on the anterior part of temporalis muscles (1cm apart from the external lateral region of the eyebrows), with the reference electrode on the sternum. The electromyographic activity was recorded during MVC, with patients seated with their head in a relaxed position. Patients were asked to clench their teeth with maximum force for 5 seconds and to repeat this process three times with a 2-minute interval rest period [15]. The complete EMG signal was captured at a frequency of 1000 Hz, followed by band-pass filtering for 20–500 Hz to obtain the root-mean-square (RMS) value pertaining to 5 seconds of MVC of the temporalis muscles. MiotecSuite software 1.0 (Miotec Equipamentos Biomédicos, Porto Alegre, Brazil) was used for data analysis, and the mean of the three MVC recordings was used for statistical analyses.

#### Self-Perception of Masseter Prominence

To assess patient’s perception about the enlargement of the lower third of their face (masseter hypertrophy), a ten-point masseter prominence photonumeric classification scale for women (Merz)[12] was used. This scale assesses masseter prominence severity from I (none) to V (much) and the position of the maximum masseter prominence (low or high) in rest and contracted positions. Patients were asked to rate their MH in all the assessment periods of the study.

#### Subjective Pain Intensity

Patients rated their subjective pain intensity in the masticatory muscles on a 0-10 cm Visual Analogue Scale (VAS) with the endpoints “no pain” and “worst imaginable pain” [16]. Participants were instructed to make a mark on the VAS indicating the current level of pain and to identify all the painful regions in a drawing of both sites of the face head and neck (at each visit).

### Statistical Analysis

Data were assessed for normality distribution using the Shapiro–Wilk test. As data for muscle thickness did not present normal distribution, data were submitted to Log10(x+1) transformation, and parametric tests were used. US, EMG, and VAS were analysed by using two-way ANOVA with repeated measures and post hoc Sidak test. Non-normally distributed photonumeric scale data were examined using Friedman’s test for within-group and Mann–Whitney test for between-group comparisons. IBM SPSS software was employed for statistical analyses, setting the significance threshold at *p* < 0.05.

## Results

**Fig. 1:**
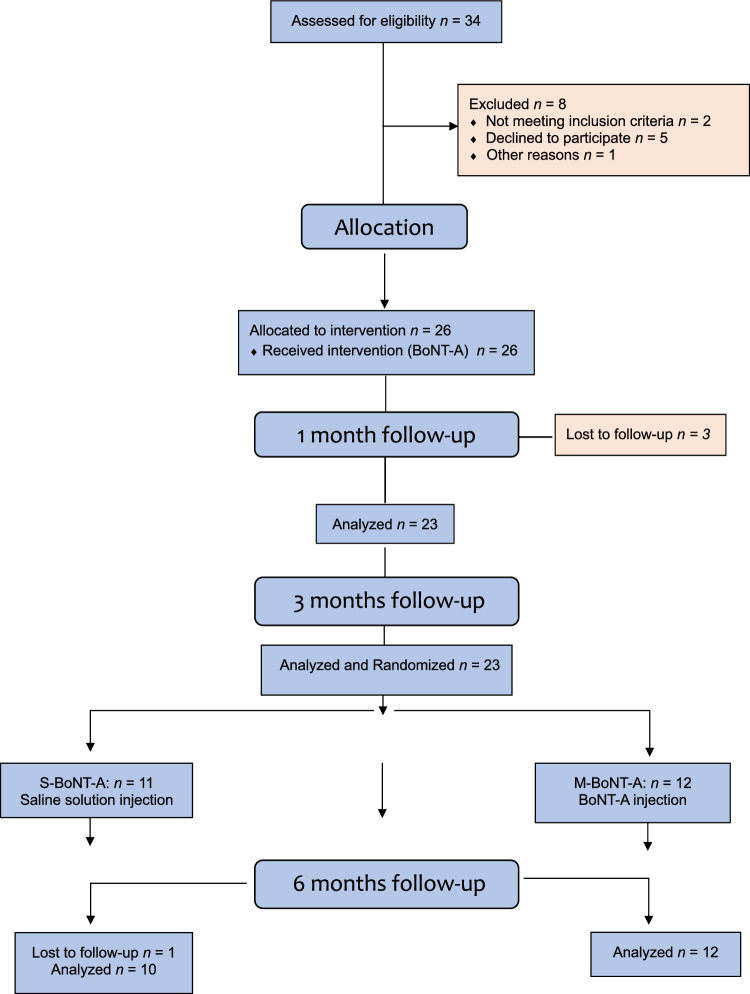
CONSORT flow diagram

The present study considered 34 patients for screening; eight did not meet the inclusion criteria, leading to the enrolment of 26 patients (32.5 ± 8.04) (Fig. 1). However, three patients withdrew from the study before the 1-month follow-up (S-BoNT-A = 2 and M-BoNT-A = 1) and one more patient before the 6-month follow-up in S-BoNT-A group, resulting in a total of 22 patients completing the study (S-BoNT-A: *n* = 10 and M-BoNT-A: *n* = 12). No significant differences regarding age were found between groups (*p* > 0.05) (Fig. 1).

### Muscle Thickness

#### Right Temporalis

**Fig. 2:**
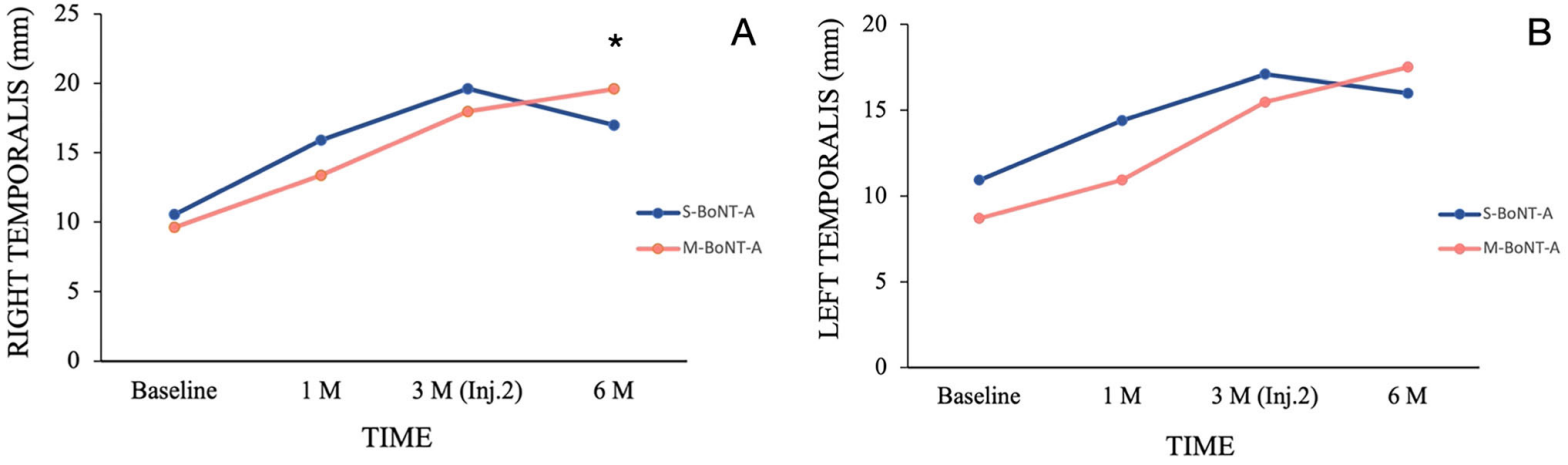
Changes in muscle thickness (mm) during maximum voluntary contraction (MVC) for each group in different time points. Inter-group differences: *p < 0.05 (right temporalis). Inj.2: Second injection session

A significant increase was observed in temporalis muscle thickness during MVC when comparing baseline with the 3-month follow-up values within S-BoNT-A and M-BoNT-A (*p* < 0.02) and when comparing baseline with the 6-month follow-up data just in M-BoNT-A group (*p* < 0.01) in the intra-group comparisons. Inter-group comparisons showed a higher muscle thickness for M-BoNT-A group at the 6-month follow-up (*p* < 0.02) (Fig. 2A).

#### Left Temporalis

Regarding intra-group comparisons, a significant increase was observed in temporalis muscle thickness in both groups comparing baseline and 3-month follow-up values (*p* < 0.01) and when comparing baseline with the 6-month follow-up data just in M-BoNT-A group (*p* < 0.001). Inter-group comparisons revealed no significant differences in temporalis muscle thickness in all follow-ups (*p* > 0.05) (Fig. 2B).

### Electromyographic Activity

#### Right Temporalis

**Fig. 3:**
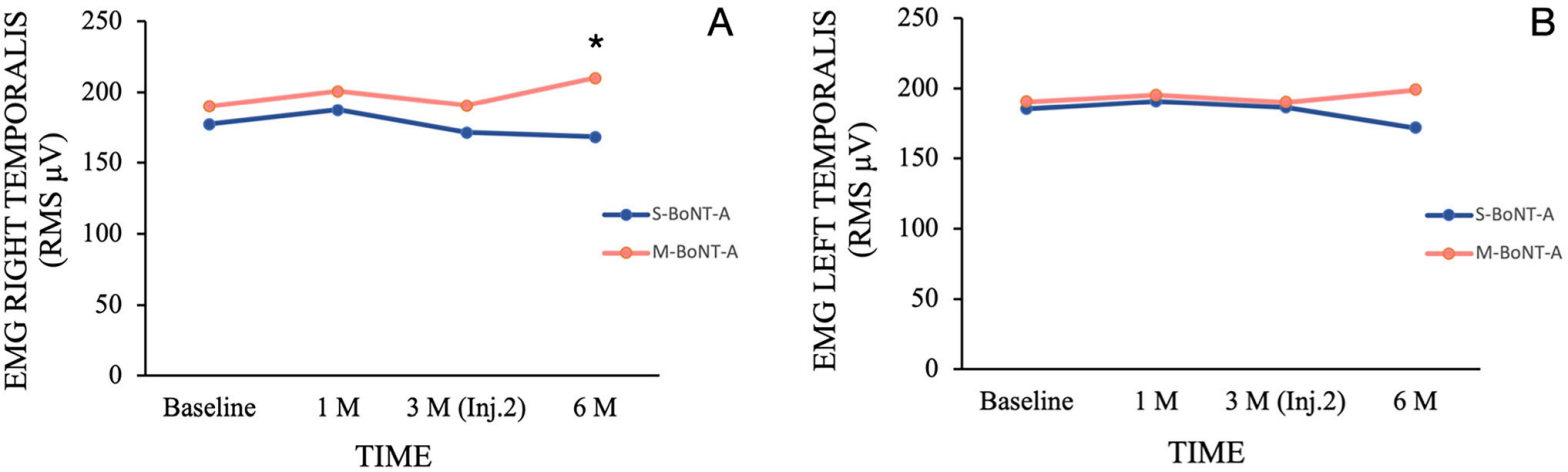
Changes in root-mean-square (RMS μV) scores in maximum volunteer contraction (MVC) for each group in different time points. Inter-group differences: *p < 0.01 (right temporalis), EMG: Electromyography; Inj.2: Second injection session

Intra-group comparisons revealed a significant raise in muscle electric activity after 6 months just for M-BoNT-A, when compared with baseline (*p* < 0.03). Conversely, no significant changes were observed in S-BoNT-A group throughout the study (*p* > 0.05). In inter-group comparisons, significant differences were observed only in the final follow-up, with higher temporalis electric activity found in M-BoNT-A (*p* < 0.01) (Fig. 3A).

#### Left Temporalis

In the intra-group comparisons, no significant changes were found in both groups through all the study (*p* > 0.05). Likewise, in the inter-group comparisons, no significant differences were found in all assessment periods (*p* > 0.05) (Fig. 3B).

### Masseter Prominence

Intra-group comparisons showed a significant decrease in MH for S-BoNT-A group just in rest position, when baseline and the 3- and 6-month follow-up data were compared (*p* < 0.03). On the other hand, a significant diminution was found for M-BoNT-A group in rest (*p* < 0.02) and contracted position (*p* < 0.0001), when comparing the baseline assessment with all the follow-ups. Regarding inter-group comparisons, a higher improvement was found just in the one-month follow-up for the rest (*p* < 0.02) and contracted positions (*p* > 0.03), in M-BoNT-A group (Table 1).

**Table 1:** Number and frequencies (%) for the ten-point photonumeric masseter prominence scale between groups at different assessment periods.

Relaxed	Baseline		1M		3M		6M	
	S-BoNT-A	M-BoNT-A	S-BoNT-A	M-BoNT-A	S-BoNT-A	M-BoNT-A	S-BoNT-A	M-BoNT-A
I	0	0	0	4 (33.3)	2 (16.7)	4 (33.3)	4 (33.3)	3 (25.0)
II	1 (8.3)	2 (16.7)	5 (41.7)	6 (50.0)	3 (25.0)	6 (50.0)	3 (25.0)	7 (58.3)
III	7 (58.3)	7( 58.3)	4 (33.3)	1 (8.3)	6 (50.0)	2 (16.7)	4 (33.3)	1 (8.3)
IV	4 (33.3)	2 (16.7)	3 (25.0)	0	1 (8.3)	0	1 (8.3)	1 (8.3)
V	0	1( 8.3)	0	1 (8.3)	0	0	0	0
*p*			*					
Contracted								
I	0	0	0	5 (41.7)	2 (16.7)	4 (33.3)	2 (16.7)	3 (25.0)
II	3 (25)	1 (8.3)	6 (50.0)	4 (33.3)	3 (25.0)	5 (41.7)	4 (33.3)	7 (58.3)
III	4 (33.3)	5 (41.7)	3 (25.0)	2 (16.7)	6 (50.0)	3 (25.0)	1 (8.3)	0
IV	4 (33.3)	4 (33.3)	3 (25.0)	1 (8.3)	1 (8.3)	0	5 (41.7)	2 (16.7)
V	1 (8.3)	2(16.7)	0	0	0	0	0	0
*p*			*					

Inter-group differences: *p < 0.02 (relaxed); *p < 0.03 (contracted)

M: month

### Subjective Pain Intensity

Baseline data showed that the mean pain intensity was 3.4±3.1 and 2.6±2.2 for S-BoNT-A and M-BoNT-A groups, respectively. Regarding intra-groups comparisons, no significant improvements were observed in both groups when compared baseline data with follow-ups (*p* > 0.05). Likewise, no significant differences were found between groups at baseline (*p* > 0.05) and when comparing all follow-ups.

Considering the painful regions of the patients, at baseline in S-BoNT-A group three patients presented pain in masseter muscle and three in temporalis muscle; also, in M-BoNT-A group, two and three patients presented pain in masseter and temporalis muscles, respectively. After the 6-month follow-up, just the M-BoNT-A group presented seven patients with pain solely in temporalis muscle. Inter-group comparisons showed a higher prevalence of painful regions in M-BoNT-A group at the 6-month follow-up (*p* < 0.02).

## Discussion

In the current randomized triple-blinded study, we assessed the effects and changes on temporalis muscles induced by both single and multiple injections of BoNT-A in the masseter muscles for MH treatment. The results of the study showed significant increase in the thickness of anterior temporalis muscle after 3 months of single or multiple injections and after 6 months just for those receiving two injections of BoNT-A. Also, EMG activity of the anterior temporalis muscle increased at the 6-month follow-up just in M-BoNT-A group. Further, as secondary findings, our study showed a significant subjective reduction of MH for both groups, with lower values for M-BoNT-A group just at the one-month assessment and a higher prevalence of painful regions at the 6-month follow-up for the same group.

The masticatory system plays a pivotal role within the intricate human anatomical framework [17]. This system comprises vital tissues and organs responsible for tasks such as chewing and digestion, and influences speech articulation, breathing, and body balance [18, 19]. Consequently, any alteration within masticatory system structures can reach implications for health across multiple domains, affecting, in addition, other components of the same system [18, 20]. In addition, any disturbance in a masticatory muscle, such as a decrease in masseter muscle mechanical activity caused by the inherent paralytic effect of BoNT-A as an aesthetic treatment for MH, may alter the operation pattern of other masticatory muscles, leading to shifts in mandibular kinematics and masticatory coordination [21]. On the contrary, studies have shown that changes in muscular coordination with BoNT-A in chewing muscles can be advantageous in managing sialorrhoea (drooling) in patients with Parkinson's disease [22], as well as in reducing muscle hypertrophy and stiffness and enhancing the facial contours and aesthetics in people with MH [23, 24]. The results of the current study showed an increase in the thickness and EMG activity of the anterior temporalis 6 months after multiple BoNT-A injections in the masseter muscle. This observation may indicate that when the masseter muscle’s contraction decreases, it may trigger compensatory enhancement in other (such as anterior temporalis) masticatory muscles’ functionality. This enhancement could potentially lead to increased muscle thickness and subsequent changes in facial shape, especially in the anterior temporalis muscle context resulting in a more oval face in patients complaining of temporal hollowing.

As far as we know, our study is the first one in reporting an increased thickness and EMG activity and development of subjective pain in temporalis muscles after BoNT-A injections in masseter muscles for MH. In a previous study, the authors found a significant increase in the temporalis volume (no thickness) in women with MH treated with BoNT-A [8]. Our muscle thickness results could not be directly compared to this study since the assessments were done by three-dimensional (3D) surface scanning for muscle volume and no thickness assessment was evaluated by ultrasound as in our study. However, it can be proposed that the increase in temporalis volume reported by the previous study is closely related to the increment of temporalis muscle thickness. As mentioned before, the interaction of the muscles of mastication is the explanation for this result. By diminishing masseter muscle contraction due to BoNT-A injections, a potential increase in temporalis volume and thickness could be expected, due to an increased compensatory contraction activity in temporalis muscle. In addition, an animal study has proved that the anterior temporalis exhibits an increase in thickness due to a compensatory mechanism for reduced function of the masseter muscles [21].

As recommended by the literature [8], our study also assessed the electrical activity of temporalis muscle after BoNT-A injections in masseter muscles and found a significant increase in the electrical activity just after 3 months of the second injection of BoNT-A. This increased activity could be a sign of adaptive changes in muscle recruitment patterns to compensate the lack of proper function of the masseter muscle, which, consequently, could generate painful symptoms in the adapted muscle. In this direction, our study found that the number of patients with pain in temporalis muscle was higher in the M-BoNT-A group, just at the 6 months assessment. The augmented contraction of the temporalis muscles could perhaps generate microtraumas in the muscle, which in some cases could cause pain [25]. It is also suggested that lengthening and increase in tension of the muscles as a result of eccentric contractions are known to cause muscle damage and delayed onset of muscle soreness resulting in pain [26]. Therefore, it is important to maintain a close control of MH patients treated with BoNT-A, in order to assess possible harmful symptoms as a consequence of repeated injections of this treatment, since such increase of painful regions was not observed in the S-BoNT-A group.

Regarding the aesthetic results, it is important to notice that even though our study found a significant improvement of masseter prominence comparing baseline values with all follow-ups, a booster injection of BoNT-A three months after the first injection did not improve the subjective aesthetic outcome since no significant differences were found between groups. Considering all our findings, we can state that there is no need to inject BoNT-A every 3 months for MH treatment, since a second injection after three months of the first one causes more adaptive changes that in long term could be detrimental for the patient, than an improvement in the aesthetic results. We recommend that future randomized clinical trials assess the clinical relevance of the adaptive changes not only in the temporal muscle, but also in other masticatory muscles.

### Study Limitations and Strengths

It is advisable to interpret the findings of this study with caution due to certain limitations that need to be addressed. Our study exclusively focused on women; thus, it is not appropriate to generalize our findings to male populations. Considering that men tend to have thicker masticatory muscles, higher doses of BoNT-A may be necessary to achieve optimal aesthetic outcomes for MH. Additionally, comparing our results to existing studies was not feasible, as no previous study has investigated the effects of BoNT-A as a treatment for MH on temporalis muscle using both ultrasound and electromyography methods. The key strength of our study lies in its meticulous methodology, particularly the randomized triple-blinded design and the longitudinal evaluation of the effects of single and multiple injections of BoNT-A, which enhances the credibility of our findings.

## Conclusion

In summary, the study suggests that repeated injections of BoNT-A for MH lead to alterations in the temporalis muscle. These changes involve variations in muscle thickness (could be considered beneficial for temporalis hollowing in patients not developing painful symptoms), EMG activity, and pain perception. It is still unknown whether these changes are irreversible; however, they must be considered as a caution for this treatment and for emphasize the concept of a full-face assessment, including function assessment, in aesthetic treatment of the face. In accordance with the fundamentals in health care, practitioners/clinicians and researchers have the responsibility to carefully assess both the benefits and risks of medical interventions. Regarding this, it should be considered whether inducing substantial structural and functional alterations in masticatory muscles through BoNT-A injections for aesthetic purposes in the masseter muscle is a prudent approach.

## Institutional Review Board Statement

The present study was approved by the Research Ethics Committee of Uningá University, Parana, Brazil (CAAE: 63135022.3.0000.5220) and the Brazilian Registry of Clinical Trials (RBR-7tdjcn5-24/01/2024). All procedures performed in this study involving human participants were in accordance with the ethical standards of the institutional and/or national research committee and with the 1964 Helsinki Declaration and its later amendments or comparable ethical standards.

## Data Availability

Datasets related to this article will be available upon reasonable request to the corresponding author.
